# The Importance of Social Support Networks on Mental Health Status of Custodial Grandparents

**DOI:** 10.1093/geroni/igab046.1002

**Published:** 2021-12-17

**Authors:** Deborah Whitley, Youjung Lee, Yanfeng Xu

## Abstract

This symposium presents a collection of papers that examine the concept of social support and its effect on custodial grandparents’ (CG) mental health state. Each paper explores a different perspective about grandparents’ access to and/or use of social support networks and mental health outcomes; several papers view social support within the context of the COVID-19 pandemic. Nadorff and colleagues explore social support by middle-generation family members and its effects on grandparents’ stress and depressive symptoms. Musil and colleagues report on psychosocial and social support predictors of self-appraised healthcare and financial security by CG during the Covid-19 pandemic. Whitley and Kelley describe current social networks relied upon by a preliminary sample of CG while managing the daily stresses and strains associated with COVID-19 and its restrictive mandates. The final two papers report the use of specialized technology and support services delivered to homebound CG during the COVID-19 pandemic. Lee and colleagues describe a telemental health model using Solution-Focused Brief Therapy to serve socially isolated grandparents experiencing mental health distress as during the pandemic. Mendoza and Park report on program challenges and outcomes of implementing a support service for grandparents living under COVID-19 restrictions. The highlights of the papers will be discussed by Yanfeng Xu and give attention to the ways scholars and practitioners can build upon these works to maximize the mental health outcomes of CG, while managing to live in socially restrictive and challenging environments.

